# Adieu Bias: Debiasing Intuitions Among French Speakers

**DOI:** 10.5334/pb.1260

**Published:** 2024-04-18

**Authors:** Nina Franiatte, Esther Boissin, Alexandra Delmas, Wim De Neys

**Affiliations:** 1 UniversitéParis Cité, LaPsyDÉ, CNRS, 46 rue Saint Jacques, 75005 Paris, France; 2 Research and Development Team, Onepoint, 2 rue Marc Sangnier, 33110 Bègles, France

**Keywords:** Reasoning, Heuristics and biases, Debiasing, Intuition, French

## Abstract

Recent debiasing studies have shown that a short, plain-English explanation of the correct solution strategy can improve reasoning performance. However, these studies have predominantly focused on English-speaking populations, who were tested with problem contents designed for an English-speaking test environment. Here we explore whether the key findings of previous debiasing studies can be extended to native French speakers living in continental Europe (France). We ran a training session with a battery of three reasoning tasks (i.e., base-rate neglect, conjunction fallacy, and bat-and-ball) on 147 native French speakers. We used a two-response paradigm in which participants first gave an initial intuitive response, under time pressure and cognitive load, and then gave a final response after deliberation. Results showed a clear training effect, as early as the initial (intuitive) stage. Immediately after training, most participants solved the problems correctly, without the need for a deliberation process. The findings confirm that the intuitive debiasing training effect extends to native French speakers.

## Introduction

Although humans have unique capacities to reason, they are often prone to cognitive biases. Most of the time, people tend to over-rely on fast, intuitive impressions rather than on more demanding, deliberative reasoning when making decisions (Evans, 2003, 2008). This intuitive or so-called ‘heuristic’ thinking can be useful in many contexts because it is fast, effortless, and often provides valid problem solutions. However, it can also conflict with the most elementary logical or probabilistic principles (e.g., Kahneman, 2011).

For instance, imagine you are analysing the results of a survey in which 1000 people took part. Of the 1000 people, 995 are Americans, and the other 5 are French. You know that one person was drawn randomly from all participants. Next, you are informed that this person loves wine, often goes on strike and has a full month of paid vacation. What do you think is most likely now: Is this person American or French? For many of us, the first response that spontaneously springs to mind is ‘a French’. This response is based on stored stereotypical associations cued by the description (e.g., ‘French are often perceived as loving wine and going on strike’). If your only piece of information were the description, this answer would probably be correct, as it is likely that there are more French than Americans who love wine and often go on strike. However, if you consider the extreme base-rate information available (995 Americans vs. 5 French), opting for the ‘American’ option becomes a more compelling choice. Yet untrained people typically neglect the base-rate principle and opt for the intuitive response that is cued by their stereotypical prior beliefs (e.g., Kahneman & Tversky, 1973).

The dichotomy between these two types of responses can be explained by the dual-process model. It characterizes human reasoning as an interplay between two types of processes or ‘systems’: A fast, intuitive one (often called ‘System 1’) and a slower, deliberative one (often called ‘System 2’; Evans & Stanovich, 2013; Kahneman, 2011). Reasoners who successfully solve the problem in line with standard logico-mathematical principles (e.g., select ‘an American’ in the above example) would correct their initial intuitive response (e.g., ‘a French’) after engaging in deliberative calculations (Morewedge & Kahneman, 2010). However, reasoners often refrain from engaging in such calculations. Instead, they default to intuitive processes without considering that the correct answer could be different (Evans & Stanovich, 2013; Kahneman & Frederick, 2005). Hence, as the base-rate example illustrates, relying on mere intuitive thinking can sometimes bias our reasoning (Evans, 2003, 2010; Stanovich & West, 2000).

In many domains, biased judgment can have detrimental impacts (e.g., policy, medicine, law, or education). Against this backdrop, reasoning scholars have long been trying to remediate people’s biased thinking (e.g., Lilienfeld et al., 2009; Milkman et al., 2009; Nisbett, 1993). Recent successful debiasing studies have shown that a short training intervention can often help people to reason more accurately. This intervention consists of a plain-English explanation about the correct solution strategy and the typical biased response to a reasoning problem (e.g., Boissin et al., 2021, 2022; Claidière et al., 2017; Franiatte et al., 2024; Hoover & Healy, 2017; Morewedge et al., 2015; Purcell et al., 2020; Trouche et al., 2014). Typically, in these studies, reasoners who received the intervention are able to produce correct responses to structurally similar problems afterwards.

These recent debiasing training results are promising. However, the nature of the training effect remains unclear. A key question is whether the training primarily affects people’s intuitive or deliberate thinking. The common assumption is that after training, participants will be more likely to deliberate properly and engage their ‘System 2’ to correct the intuitively generated heuristic response (e.g., Evans, 2019; Lilienfeld et al., 2009; Milkman et al., 2009). This idea aligns with the ‘corrective’ dual-process view which posits that the deliberate ‘System 2’ primarily serves to correct the intuitive ‘System 1’ (Kahneman, 2011). However, in theory, it is also possible that once reasoners grasp the solution after the problem is explained, they will no longer generate an incorrect intuitive response. Instead, they might apply the correct solution strategy intuitively without the need for a corrective ‘System 2’ deliberation process.

Recent evidence provided some support for the ‘trained intuitor’ viewpoint (e.g., Boissin et al., 2021, 2022). These studies used a two-response paradigm (Thompson et al., 2011) to determine whether the explanation affected participants’ intuitive and/or deliberate reasoning. In this paradigm, participants are asked to give two consecutive responses to a reasoning problem. First, they have to respond as fast as possible with the first intuitive hunch that comes to mind. Next, they can take all the time they want to reflect on the problem and give a final response. To make sure that the initial answer is generated intuitively, people have to respond under time pressure and, at the same time, perform a secondary memory task that burdens cognitive resources and disrupts the potential involvement of the deliberative ‘system’ (Bago & De Neys, 2019). Two-response findings indicate that while the majority of reasoners are biased before the training (both at the initial and final response stages), immediately after receiving the explanation, most of them are able to provide correct responses. Critically, their responses are correct as early as the initial, ‘intuitive’ stage. This suggests that the debiasing approach allows people to intuit correctly rather than to boost their deliberate correction.

Given that the ‘trained intuitor’ debiasing approach has important applied and theoretical implications, further validation is needed. However, a critical limitation of (debiasing) training studies is their predominant focus on (native) English speakers, who were tested with problem contents designed for an English-speaking test environment (e.g., Boissin et al., 2021, 2022, 2023a; Franiatte et al., 2024; Hoover & Healy, 2017; Morewedge et al., 2015; Purcell et al., 2020). Such a limited scope of (most) psychological studies has been questioned by various scholars: They pointed out the lack of representation of diverse populations and languages, urging for greater inclusivity in scientific research (e.g., Arnett, 2008; Blasi et al., 2022; Huettig & Ferreira, 2023; Thalmayer et al., 2021). More specifically, critics have put forward the theoretical and practical limitations stemming from the Anglocentric bias: For instance, the overemphasis on features and mechanisms that are specific to English-speaking people over other populations (see Blasi et al., 2022). Numerous studies also pointed to the overlooked structural differences between languages (e.g., Evans & Levinson, 2009). They can have consequences for other aspects of cognition, ostensibly non-linguistic, such as causal cognition (Bender & Beller, 2019) or biased cognition (Smith et al., 2018).

This critical language limitation raises concerns about the generalizability of recent debiasing findings. Arguably, if we want to guarantee the robustness of the debiasing approach and findings, it seems important to broaden the scope of our research to other languages and cultural settings. As a first step, in the present study, we will test whether the keystone results of previous debiasing studies can be extended to native French speakers living in continental Europe (France).

We used the exact same training procedure and problem test battery as in previous debiasing work (see Franiatte et al., 2024). The only difference was that our participants were native French speakers and all our problem content was adapted to French. The test battery consisted of three popular classic reasoning tasks: The base-rate neglect (Kahneman & Tversky, 1973), conjunction fallacy (Tversky & Kahneman, 1983), and bat-and-ball tasks (Frederick, 2005). They were combined in a one-hour training battery. For each task, the training consisted of three different blocks: A pre-intervention, an intervention, and a post-intervention. Participants were randomly assigned to a training or control group. In the intervention block, participants from the training group solved task problems and always received a short debiasing explanation about the rationale behind the task, while participants of the control group simply solved the problems without receiving the explanation. During the pre- and post-intervention blocks, we used the two-response paradigm to determine whether the intervention affected participants’ intuitive and/or deliberate reasoning.

## Method

### Preregistration and data availability

The study design and research questions were preregistered on the AsPredicted website (https://aspredicted.org) and stored on the Open Science Framework. No specific analyses were pre-registered. Raw data, analysis script, and preregistration are also available on the Open Science Framework (https://osf.io/hk8rv/).

### Participants

Participants were volunteers and were recruited online through several communication channels in continental Europe (France).^1^ They were not paid for their participation. Only native French speakers were allowed to take part in the study.

In total, 147 reasoners participated in the study (71 females, *M*_age_ = 37.6 years, *SD* = 12.4), 70 participants were randomly assigned to the training group and 77 to the control group. Among them, 12 had secondary school as their highest level of education, and 135 reported a university degree.

We aimed to recruit a minimum of 100 subjects. Our sample size decision was based on Boissin et al.’s (2021) original study, who tested 100 participants. The experiment ran for three months in the summer of 2022 (early June to late August). We decided to include all participants who had completed the study during that time window. This allowed us to detect small-to-medium training effect (*d* = .41) between the pre- and post-intervention blocks with a power of 80%. All reported results and analyses concern the 147 participants who completed the study.

### Materials

The test session was composed of three different reasoning tasks (i.e., base-rate neglect, conjunction fallacy, and bat-and-ball tasks). In each session, for each participant, the task order was randomized. Each task contained eight conflict and eight no-conflict problems (see further) and was composed of three blocks presented in the following order: A pre-intervention, a short intervention, and a post-intervention block. In total, each participant had to solve 48 problems. All these problems had been adapted in French before running the experiment (see Procedure). Problems are presented in Supplementary Material Section A. For convenience, we will always illustrate the problem content in the main text with English examples.

#### Base-rate neglect problems (BR)

Each participant was presented with base-rate problems based on Pennycook et al. (2014) that were already adapted in French by Boissin et al. (2023b). Participants always received a description of the composition of a sample (e.g., ‘This study contains writers and construction workers’), a description that was designed to cue a stereotypical association (e.g., ‘Person ‘W’ is strong’), and a base rate information (e.g., ‘There are 996 writers and 4 construction workers’). Participants’ task was to indicate to which group the person most likely belonged. The task instructions stressed that the person was drawn randomly from the specified sample. The problem presentation format was based on Pennycook et al.’s (2014) rapid-response paradigm. The base rates and descriptive information were presented serially, and the amount of text presented on screen was minimized. As in Pennycook et al. (2014), base rates varied between 995/5, 996/4, and 997/3. We labelled the response that is in line with the base rates as the correct response (see Supplementary Material Section A). Table 1 illustrates the full problem format.

**Table 1:** Examples of conflict and no-conflict problems for the three reasoning tasks used in the battery: Base-rate neglect, Conjunction Fallacy, and Bat-and-ball. For convenience, the problem content is illustrated in English. The corresponding French material can be found in the Supplementary Material Section A.


	CONFLICT VERSION	NO-CONFLICT VERSION

**Base-rate neglect**	This study contains writers and construction workers. Person ‘W’ is strong. There are 996 writers and 4 construction workers. Is Person ‘W’ more likely to be: A writer A construction worker	This study contains writers and construction workers. Person ‘W’ is strong. There are 996 construction workers and 4 writers. Is Person ‘W’ more likely to be: A writer A construction worker

**Conjunction Fallacy**	Kadin, 32, has previously studied astronomy and likes sci-fi. Is it most probable that the described person is: A longshoreman A longshoreman and a stargazer An Oscar winner A longshoreman and an equestrian	Kadin, 32, has previously studied astronomy and likes sci-fi. Is it most probable that the described person is: A stargazer A longshoreman and a stargazer An Oscar winner A longshoreman and an equestrian

**Bat-and-ball**	In a park, there are 140 adults and children in total. There are 100 more adults than children. How many children are there? 40 children 10 children 60 children 20 children	In a park, there are 140 adults and children in total. There are 100 adults. How many children are there in the park? 40 children 10 children 60 children 20 children


To ensure that possible correct or incorrect responses did not originate from guessing, we also presented no-conflict control problems. In these control problems, the description always triggered a stereotypical trait of a member of the largest group. The heuristic intuition thus cued the correct response. Participants had to select the correct response among the same two answer options as for a corresponding standard conflict version (see Table 1).

We presented four conflict and four no-conflict problems in the pre- and post-intervention blocks. These no-conflict problems should be easy to solve. If participants are paying minimal attention to the task and refrain from random guessing, they should show high accuracy (Bago & De Neys, 2019).

#### Conjunction fallacy problems (CF)

Each participant was also presented with conjunction fallacy problems. Our item material was based on a new pilot study in which we adapted and pretested conjunction fallacy problems in French (see *Pilot rating study* in Procedure). We used the conjunction task format introduced by Andersson et al. (2020): All conjunction problems presented a short personality description of a character, consisting of their name (e.g., ‘Kadin’), their age (e.g., ‘32’), their previous studies (e.g., ‘astronomy’) and their hobby/interest (e.g., ‘sci-fi’). Next, the participants were given four response options and were asked to indicate which one was most likely. In the critical conflict problems, one option presented a characteristic that featured an unlikely stereotypical association given the description (e.g., ‘a longshoreman’), and one option presented a conjunction of this unlikely and a likely characteristic (e.g., ‘a longshoreman and a stargazer’). Two other filler options presented a very unlikely characteristic (e.g., ‘an Oscar winner’) and a conjunction of two unlikely characteristics (e.g., ‘a longshoreman and an equestrian’). Table 1 illustrates the full problem format.

We also presented four conflict and four no-conflict control problems in the pre- and post-intervention blocks. In the no-conflict control problems, we replaced the singular unlikely response option with the option that featured the likely stereotypical association (e.g., ‘a stargazer’ in the above example, see Table 1). Reasoners will tend to select the statement that best fits with the stereotypical description (Tversky & Kahneman, 1983). The fit will be higher for the likely than the unlikely characteristic with the conjunctive statement falling in between. Hence, on the no-conflict problems, stereotypical associations will no longer favour the conjunctive over the singular statement and participants are expected to show high accuracies (see De Neys et al., 2011).

The four response options were presented in random order. Note that Andersson et al. (2020) adopted the four options design to minimize the use of simple visual response strategies (e.g., ‘always choose the shortest answer’). As in the Andersson et al. study, selection of the filler options was overall low in our study (i.e., 17.4% of options). However, strictly speaking, participants who select the singular very unlikely option (e.g., ‘an Oscar winner’ in the above example) do not violate the critical conjunction rule. As Boissin et al. (2022) mentioned, given that we are interested in learning effects, selection of the very unlikely option can be considered a correct response. Hence, we considered answers on which the conjunction fallacy is avoided (i.e., unlikely and very unlikely answers) as correct answers. Figure S1 in Supplementary Material Section B gives a detailed overview of the selection frequency of each individual response option.

#### Bat-and-ball problems (BB)

We also presented problems taken from Raoelison and De Neys (2019). They were modified, French versions of the original bat-and-ball problem (Frederick, 2005), which used quantities instead of prices (e.g., ‘*In a park there are 140 adults and children in total. There are 100 more adults than children. How many children are there?’)*. Participants had to select the correct response among four response choices which were composed of (1) the correct response (i.e., ‘20 children’ in the above example), (2) the intuitively cued ‘heuristic’ response (i.e., ‘40 children’ in the above example), (3) a foil option which was the sum of correct and heuristic answers (i.e., ‘60 children’), and (4) a second foil option which was the second greatest common divider (i.e., ‘10 children’). Mathematically speaking, the correct equation to solve the above bat-and-ball problem is: ‘100 + 2x = 140’. Instead, people are thought to be intuitively using the ‘100 + x = 140’ equation to determine their response (Kahneman, 2011). The latter equation was used to determine the ‘heuristic’ answer option, and the former to determine the correct answer option for this problem. The four response choices appeared in random order. Table 1 illustrates the full problem format.

We also presented four conflict and four no-conflict control problems in the pre- and post-intervention blocks. In the no-conflict control problems, the conflict was removed by deleting the critical relational ‘more than’ statement. The heuristic intuition thus cued the correct response (see Table 1; De Neys et al., 2013). In this case, the intuitively cued ‘40 children’ answer was correct. Note that, as Boissin et al. (2021), we added three words to the control problem questions to equate the semantic length of the conflict and no-conflict versions. Participants had to select the correct response among the same four answer options as for a corresponding standard conflict version. As in the other tasks, these control problems should be easy to solve: If participants are paying minimal attention to the task and refrain from random guessing, accuracy should be at ceiling (Bago & De Neys, 2019).

#### Counterbalancing

For every reasoning task, two sets of problems were created in which the conflict status of each problem (see above) was counterbalanced. More specifically, all the conflict problems of the first set appeared in their no-conflict version in the second set, and vice-versa. Half of the participants were presented with the first set of problems, while the other half was presented with the second set. Hence, in each task, the same content was never presented more than once to a participant, and everyone was exposed to the same problems, which minimized the possibility that mere problem differences influence the results. The presentation order of the tasks and the problems within each task was also randomized.

#### Intervention block

In the intervention block, participants had to solve three additional conflict problems (i.e., three base-rate or three conjunction fallacy or three bat-and-ball problems depending on the task), without any cognitive or time constraint. In the training group, participants were explained the correct solution after having responded to each problem, whereas in the control group, participants only responded to the problem without receiving any explanation. The explanations were translated from English to French. They were based on the same general principles that were adopted by Boissin et al. (2021, 2022): They were as brief and simple as possible to prevent fatigue or disengagement from the task. Each explanation explicitly stated both the correct response and the typical biased, incorrect response. No personal performance feedback was given to avoid promoting feelings of judgment (Trouche et al., 2014). Finally, to avoid inducing mathematical anxiety, the explanation never mentioned a formal algebraic equation (Hoover & Healy, 2017). The following example illustrates a typical question and explanation for a bat-and-ball problem:

‘Question:

*A banana and an apple cost $1.40 in total. The banana costs $1.00 more than the apple. How much does the apple cost?*

Explanation:

*The correct response is 20 cents. Many people are tempted to answer 40 cents, but this is wrong*.

*If the apple costs 40 cents, the banana would cost $1.40 (as it costs one dollar more than the apple); both together, they would then cost $1.80*.

*However, the problem said they cost $1.40 together*.

*The correct answer is that the apple costs 20 cents, the banana $1.20 so together they cost $1.40 ($0.20 + $1.20 = $1.40)*.’

#### Two-response format

We used the two-response paradigm (Thompson et al., 2011) for the presentation of all problems in the pre- and post-intervention blocks. In this paradigm, participants are asked to provide two consecutive responses on every trial: A ‘fast’ response, directly followed by a second ‘slow’ response. This method allowed us to capture both an initial ‘intuitive’ response, and then a final ‘deliberate’ one. To minimize the possibility that deliberation was involved in producing the initial ‘fast’ response, participants had to provide their initial answer within a strict time limit while performing a concurrent cognitive load task (e.g., Bago & De Neys, 2017, 2019). The cognitive load task was based on the dot memorization task (Miyake et al., 2001) given that it had been successfully used to burden executive resources during reasoning tasks (e.g., De Neys, 2006; Franssens & De Neys, 2009). Participants had to memorize a complex visual pattern (i.e., a 3 × 3 grid in which 4 dots were placed) that was presented briefly before each reasoning problem. After their initial ‘intuitive’ response to the problem, participants were shown four different matrixes, and they had to choose the correct pattern (see De Neys, 2006, for more details). They received feedback as to whether they chose the correct or incorrect pattern.

For all base-rate problems, a time limit of 3 seconds was chosen for the initial response, based on previous pre-testing that indicated it amounted to the time needed to read the preambles, move the mouse, and click on a response option. Similarly, the time limit was set to 5 seconds for conjunction fallacy problems and 8 seconds for bat-and-ball problems. For all tasks, previous pretesting established that the time limits imposed a stringent time pressure that forced participants to respond significantly faster than in a traditional unconstrained, one-response test format (Bago & De Neys, 2017, 2019; Boissin et al., 2022). Note that the time limit and cognitive load were only applied during the initial response stage and not during the subsequent final stage in which participants were allowed to deliberate.

#### Justification

For every reasoning task, after the last problem of the post-intervention block – which was always a conflict problem – participants were asked to select a rationale for their final response (they could choose between: ‘*I did the math*’ / ‘*I guessed*’ / ‘*I decided based on intuition or gut feeling*’ / ‘*Other*’). For the ‘*Math*’ and ‘*Other*’ options, they were asked to type-in an explanation for their justification. Previous work (e.g., Bago & De Neys, 2019; Boissin et al., 2021) indicated that correct reasoners typically manage to correctly justify their answers.

As in previous studies, results indicated that, for the three tasks, the majority of correct responses was correctly justified after training (training group: 105 correct justifications out of 170 correct responses, i.e., 61.8%; control group: 64 correct justifications out of 96 correct responses, i.e., 66.7%). The interested reader can find details in Table S1 in Supplementary Material Section C. Note that the justification was untimed and retrospective. It was collected for exploratory purposes and does obviously not allow drawing any conclusions regarding the intuitive or deliberate nature of participants’ processing.

### Procedure

#### Pilot rating study

The material for the base-rate and bat-and-ball task items was already adapted to French and validated in previous pilot studies (e.g., Boissin et al., 2023b; Raoelison et al., 2021). For the conjunction task, we created a pool of 52 potential French items that contained translated and culturally adapted items from Andersson et al. (2020) and newly generated items that respected the same structure. To validate the stereotypical problem content, we ran a pilot rating study with 90 participants (45 females, 2 neutral-gender, *M*_age_ = 30.2 years, *SD* = 9.8). Participants were asked to rate how well each option matched the described person on a scale from 0 (not at all similar) to 10 (very similar). To select the most appropriate material, after an initial exploration, we picked items for which, in the conflict version, the combination of the unlikely and likely constituent was rated at a minimum of 3.5 and was rated higher than the unlikely constituent. In their no-conflict counterpart, we picked items for which the likely constituent was rated at a minimum of 5 and higher than the combination of the unlikely and likely constituent. In addition, the relative option ranking needed to be maximally respected (e.g., very unlikely < unlikely < likely and unlikely combination < likely). We selected 35 items for which these differences were greatest. Among the ultimately selected items, the average ratings for the different response options were: Very unlikely option (*M* = 0.6, *SD* = 0.7); unlikely option (*M* = 1.6, *SD* = 1.5); unlikely and unlikely option (*M* = 1.3, *SD* = 1.3); unlikely and likely option (*M* = 5.1, *SD* = 1.8); and likely option (*M* = 7.1, *SD* = 1.6). In total, the 35 items were distributed as follows: 2 counterbalanced sets of 8 items in each pre- and post-intervention blocks and 3 items in the intervention block. The full item set can be found in Supplementary Material Section A.

#### Main study

The experiment was conducted online using the Qualtrics platform (https://www.qualtrics.com), either in small groups in the presence of an experimenter or at home. The procedure was similar to Franiatte et al. (2024). First, participants were instructed that the experiment would take around fifty-five minutes and that it demanded their full attention. They were told they would need to solve different types of reasoning tasks for which they would have to provide two consecutive responses. They were specifically instructed that we were interested in their very first, initial answer that comes to mind and that – after providing their initial response – they could reflect on the problem and take as much time as they needed to provide a final answer. At the beginning of each task, to familiarize themselves with the two-response procedure, they solved two unrelated practice reasoning problems. Next, they familiarized themselves with the cognitive load task by solving two load trials and, finally, they solved two problems which included both cognitive load and the two-response procedure.

**Figure 1:**
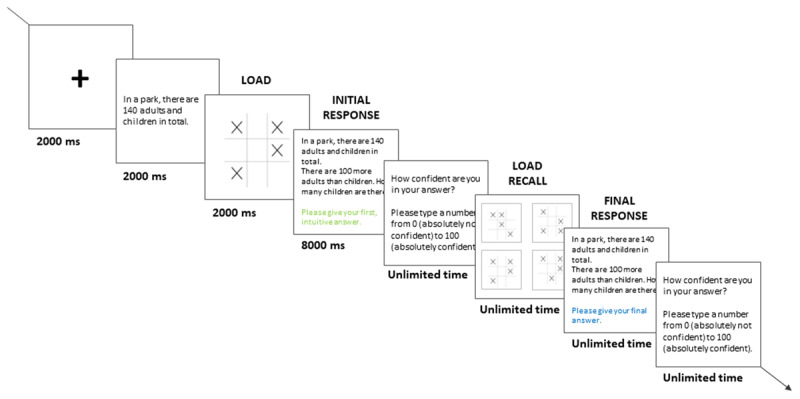
Time course of a typical two-response trial, with a bat-and-ball problem. For convenience, the problem content is illustrated in English.

Figure 1 shows a typical trial, which consisted of, first, presentation of a fixation cross displayed during 2000 ms, followed by the first sentence of the problem displayed for 2000 ms (e.g., ‘*In a park, there are 140 adults and children in total*’ for the bat-and-ball task), and followed by the visual matrix for the cognitive load task for 2000 ms. Then, the full problem was presented, at which point participants had 3000 ms (base-rate neglect), 5000 ms (conjunction fallacy), or 8000 ms (bat-and-ball) to give their initial answer. Note that in this initial ‘intuitive’ response stage, the background of the screen turned yellow after 2000 ms (base-rate neglect), 3000 ms (conjunction fallacy), or 6000 ms (bat-and-ball) to warn participants that they only had a short amount of time left to answer. If they had not provided an answer before the time limit, they were given a reminder that it was important to provide an answer within the time limit on subsequent trials. Participants were then asked to enter their confidence in the correctness of their answer on a scale from 0% (absolutely not confident) to 100% (absolutely confident). Then, they were presented with four visual matrix options and had to choose the one that they had previously memorized. Finally, the same reasoning problem was presented again, and participants were asked to provide a final ‘deliberate’ answer (without time limit nor cognitive load) and, once again, to indicate their confidence level. Note that due to a coding error the confidence data was not systematically recorded and was not further analysed (the non-missing data is included in our data file, and an exploratory analysis of the partial data can be found in Supplementary Material Section D).

At the end of the study, participants in the control group were also presented with the explanations about how the base-rate neglect, conjunction fallacy, and bat-and-ball problems could be solved, and all participants were asked to complete a page with demographic questions.

### Trial exclusion

Following our preregistration, we discarded trials in which participants failed to provide their initial answer before the deadline (5.5% of all trials) or failed to pick the correct matrix in the cognitive load task (9.6% of the remaining trials), and we analysed the remaining 90.4% of all trials. On average, each participant contributed 40.9 (*SD* = 5.1) conflict trials out of 48, and 41.0 (*SD* = 4.3) no-conflict trials out of 48.

Note that as part of our procedure, we asked participants whether they were familiar with the original bat-and-ball problem and asked them to solve it (Frederick, 2005). In total, 95 participants out of 147 (64.6%) reported having come across the problem before. Traditionally, these participants are removed from the analyses to eliminate the possibility that their prior knowledge of the correct solution affects the results (e.g., Bago & De Neys, 2019; Boissin et al., 2021). First, we ran all analyses while including these 95 participants, and second, while not including them. None of our conclusions were affected either way, and the tendencies remained the same. Thus, in line with our preregistration, we included these participants in the reported analyses in the main text (see Figure S2 in Supplementary Material Section E for overview analyses with and without these participants).

### Composite measure

For simplicity and to maximize power, our analyses focused on the composite conflict accuracy across the three different reasoning tasks (i.e., base-rate neglect, conjunction fallacy, and bat-and-ball). To calculate the composite performance, we averaged for each participant the proportion of correct initial and final responses, separately for each task. Then we averaged across all tasks (separately for initial and final trials). For completeness, we calculated the composite performance also for no-conflict trials (see Table S3 in Supplementary Material Section F).

### Statistical analyses

The data were processed and analysed using the R software (R CoreTeam, 2017) and the following packages (in alphabetical order): dplyr (Wickham et al., 2020), ggplot2 (Wickham, 2016), lmerTest (Kuznetsova et al., 2017) and tidyverse (Wickam et al., 2024).

The study uses a between-subject group variable based on the assigned group during the experiment (i.e., training or control). We measured the percentage of correct responses – referred to as the accuracy in the main text – on conflict and no-conflict items, at both initial ‘intuitive’ and final ‘deliberate’ response stages, before (pre-intervention) and after (post-intervention) receiving the text training.

Throughout the article, we used mixed-effect regression models with random intercepts and slopes for both participants and stimuli (i.e., items; see Brysbaert & Debeer, 2023). The Wald test assessed the statistical significance of the fixed effect of the model.

## Results

### Conflict trial accuracy

**Figure 2:**
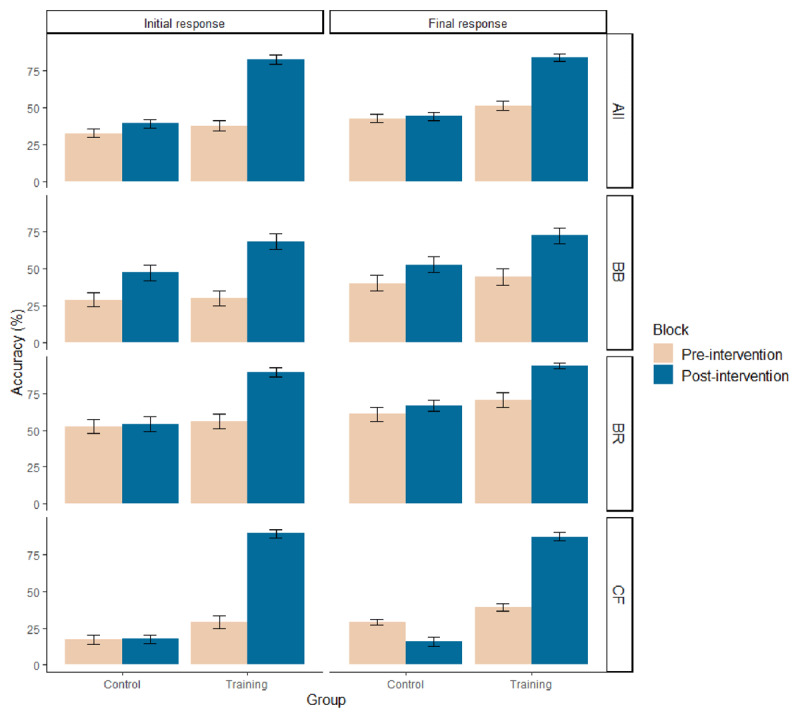
Mean accuracy (%) of correct initial and final responses on conflict problems for control and training groups, before and after the intervention, for each task (BB, BR, CF), and combined (All). Error bars are standard errors. BB = bat-and-ball, BR = base-rate neglect, CF = conjunction fallacy tasks, All = the composite mean across the three tasks.

For each task and for each participant, we analysed the average proportion of correct initial and final responses for all the conflict items, in each of the two blocks (pre- and post-intervention). First, before the intervention, participants were mostly biased and showed low initial accuracies (training group: *M* = 37.8%, *SD* = 28.2; control group: *M* = 32.8%, *SD* = 24.5; see Figure 2). The overall performance of both groups improved following the intervention. However, the accuracy increase was significantly higher in the training group (+45.0 points, *M* = 82.8%, *SD* = 24.2) than in the control group (+6.4 points, *M* = 39.2%, *SD* = 25.5). Statistical composite analyses revealed that the Block × Group interaction significantly improved the model for the initial responses, χ^2^ (1) = 103.2, *p* < .001.

In the same vein, participants showed lower final accuracies before the intervention (training group: *M* = 51.5%, *SD* = 25.5; control group: *M* = 42.8%, *SD* = 25.2) than after. In the post-intervention block, participants of the training group sharply improved their performance (+32.7 points, *M* = 84.2%, *SD* = 21.7), while those of the control group hardly improved (+1.4 points, *M* = 44.2%, *SD* = 26.0). Similarly, statistical composite analyses revealed that the Block x Group interaction significantly improved the model for the final responses, χ2 (1) = 88.2, *p* < .001. The interested reader can find details of the main effects in Tables S4 and S5 (Supplementary Material Section G).

For completeness, Figure 2 (bottom panels) shows the data for each individual reasoning task. By and large, similar initial and final response tendencies were observed for each individual task. If anything, as previously found in Franiatte et al. (2024), the training effect tended to be somewhat less pronounced for the base-rate task. However, in this task, participants’ pre-intervention performance was also already higher than for the others.

Additionally, we conducted an exploratory analysis to compare our study’s reasoning performance with the similar debiasing study—adopting the same tasks and design—conducted by Franiatte et al. (2024) on native English speakers. Overall, we observed a similar training effect in the two samples (see Figure S3 in Supplementary Material Section H). In the training group, initial responses significantly improved by 45% in the current study, and 42% in Franiatte et al.’s (2024) study. Similarly, the final responses significantly improved by 33% in the current study and 44% in Franiatte et al.’s (2024) study. Tendencies for the individual reasoning tasks were also highly similar (see Supplementary Material Section G).

In sum, in our study, we replicated previously established findings in a native French-speaking sample of reasoners. Our results are consistent with the recent debiasing literature (e.g., Boissin et al., 2021, 2022; Franiatte et al., 2024; Purcell et al., 2022) and confirm that a single, short intervention can significantly increase both initial and final response accuracy on classic reasoning tasks.

### Direction of change

**Figure 3:**
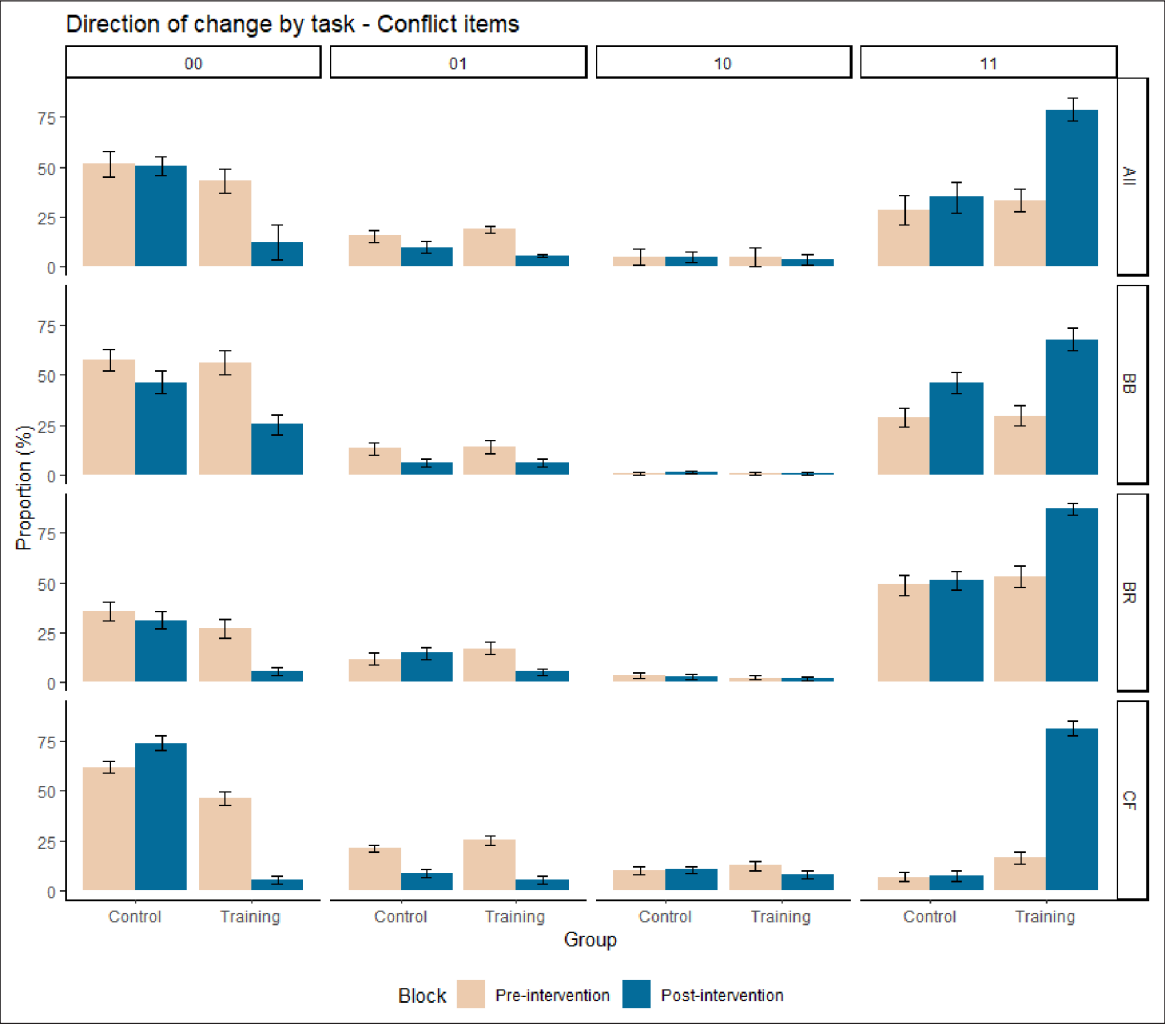
Proportion (%) of each direction of change (i.e., ‘00’ pattern, ‘01’ pattern, ‘10’ pattern, and ‘11’ pattern; 0 = incorrect response, 1 = correct response, first digit = initial response, second digit = final response) on conflict problems for control and training groups, before and after the intervention, for each task (BB, BR, CF), and combined (All). Error bars are standard errors. BB = bat-and-ball, BR = base-rate neglect, CF = conjunction fallacy tasks, All = the composite mean across the three tasks.

To gain some insight into how people changed (or did not change) their answers after deliberation, we performed a direction of change analysis for the conflict items (Bago & De Neys, 2017). Specifically, each trial is composed of two responses, the initial ‘intuitive’ one (given under time pressure and cognitive load) and the final ‘deliberate’ one. Correct responses are labelled ‘1’ and incorrect responses are labelled ‘0’. Hence, each trial can result in one of four different patterns: ‘00’ pattern, incorrect response at both response stages; ‘11’ pattern, correct response at both response stages; ‘01’ pattern, initial incorrect and final correct responses; and ‘10’ pattern, initial correct and final incorrect responses. Figure 3 plots the direction of change distribution for each block (pre- and post-intervention) and each group (control and training).

Consistent with the overall accuracies presented above, before the intervention, around half of the trials were incorrect at both response stages and produced ‘00’ (biased) patterns (training group: *M* = 43.0%, *SD* = 27.1; control group: *M* = 51.1%, *SD* = 26.1). After the intervention, similar results were observed for participants in the control group, with ‘00’ (biased) patterns remaining stable (0.1 point decrease, *M* = 51.0%, *SD* = 27.07 in the post-intervention block). However, in the training group, the intervention led to a sharp decrease in ‘00’ patterns (31.5 points decrease, *M* = 11.5%, *SD* = 18.8). Notably, the decrease in ‘00’ patterns led to a considerable increase in ‘11’ patterns (41.9 points rise in the training group vs. 6.1 points rise in the control group) rather than in ‘01’ patterns wherein we observed the opposite trend (13.5 points decrease in the training group; 5.9 points decrease in the control group). Eyeballing Figure 3 (bottom panels) indicates that we observed similar tendencies for each of the individual reasoning task. These tendencies were again highly consistent with the original Franiatte et al.’s study with English speakers (see Table S6 in Supplementary Material Section I for full details).

In sum, these results confirm that the training improved reasoning performance, as early as the initial ‘intuitive’ stage. In this study, we replicated the sound intuiting effect found in previous debiasing studies (e.g., Boissin et al., 2021, 2022). In other words, after the training intervention, reasoners were able to intuit the correct solution strategy and typically no longer required to correct an initial ‘erroneous’ response through deliberation.

### Individual level direction of change

To gain some deeper insight into how a given reasoner changed (or did not change) their response, we also performed an individual level accuracy analysis on the conflict trials (Raoelison & De Neys, 2019). For each of the 147 participants, on each conflict trial, from start to end of the experiment, we focus on their dominant direction of change and classified it using the categories introduced by Boissin et al. (2021, 2022).

First, ‘biased responders’ did not benefit from the intervention and provided a majority of incorrect responses (‘00’ trials) in pre- and post-intervention blocks. Mirroring the overall accuracy effects, they represented 55.6% of reasoners in the control group but only 15.8% of reasoners in the training group. Second, ‘correct responders’ provided a stable majority of correct answers (‘01’ or ‘11’ trials) before and after the training intervention, and thus did not require any intervention to respond correctly. They represented 31.2% of reasoners in the training group and 22.7% in the control group. Third, ‘improved responders’ are those whose accuracy increased after the training intervention. They either gave a majority of biased responses (‘00’ trials) before the intervention and then switched to a majority of correct responses after the intervention (‘01’ or ‘11’ trials), or already gave a majority of correct final responses (‘01’ trials) before the intervention but then switched to a majority of correct initial and final responses (‘11’ trials) after the intervention. They amounted to 50.5% of reasoners in the training group and 16.0% in the control group. Participants who gave inconsistent response patterns and could not be classified were put in the ‘Other’ category (2.5% in the training group, 5.8% in the control group; see Figure S4 in Supplementary Material Section J for full results).

### No-conflict trial accuracy

For completeness, for each task and each participant, we also calculated the average proportion of initial ‘intuitive’ and final ‘deliberate’ responses for all the no-conflict items. Results showed that performance was consistently at ceiling in pre- and post-intervention blocks for initial responses (*M* = 90.5%, *SD* = 12.7 in the training group; *M* = 89.1%, *SD* = 11.6 in the control group), and final responses (*M* = 92.8%, *SD* = 11.2 in the training group; *M* = 90.5%, *SD* = 10.5 in the control group). The high initial and final performance on the no-conflict control problems provides evidence against a general systematic guessing confound (Bago & De Neys, 2017). In other words, if participants were not paying attention and were simply guessing throughout the study, they should have performed much worse on the no-conflict items. It also argues against a ‘reversed heuristic’ training account (Boissin et al., 2022) in which training would simply lead participants to distrust the intuitively cued response. If this were the case, we would expect a significant decline in post-intervention no-conflict trial performance (in which the intuitive, heuristic response was always correct). A detailed overview of the no-conflict problem accuracies by task can be found in Table S3 in Supplementary Material Section F.

## General Discussion

In the present paper, we explored the debiasing effect of a short training that provides explanations for three different reasoning tasks (i.e., base-rate neglect, conjunction fallacy, and bat-and-ball tasks) in a French-speaking population. Especially, we explored whether we replicated previous debiasing findings observed in English-speaking populations (e.g., Boissin et al., 2021, 2022; Claidière et al., 2017; Franiatte et al., 2024; Hoover & Healy, 2017; Morewedge et al., 2015; Purcell et al., 2020; Trouche et al., 2014). We used a two-response paradigm to track participants’ initial ‘intuitive’ and final ‘deliberate’ responses.

Results indicated that the debiasing intervention led to a clear training effect. At the end of the session, a majority of trained reasoners were able to produce correct responses to structurally similar problems afterwards. Interestingly, the two-response findings indicated that this effect was observed as early as the initial ‘intuitive’ stage (overall 45% increase). That is, after training, reasoners no longer required correction of their erroneous intuitively generated heuristic response. Instead, they were able to produce intuitive responses consistent with logico-mathematical principles from the outset. In other words, the training intervention manages to get the majority of biased reasoners to intuit correctly. In this sense, the current study points to similar conclusions to previous debiasing studies conducted on English-speaking populations (e.g., Boissin et al., 2021, 2022; Franiatte et al., 2024; Hoover & Healy, 2017, Purcell et al., 2021).

Although we successfully replicated the training effect reported in the literature, there are also some notable differences between our study and previous debiasing findings that should be highlighted. A first thing to note is that participants in our study consistently outperformed Franiatte et al.’s (2024) reasoners in terms of accuracy. Specifically, in the French-speaking training group, before the intervention, we found a 10% higher initial accuracy and a 19% higher final accuracy compared to Franiatte et al.’s (2024) study. Similarly, after the intervention, we observed a 13% higher initial accuracy and a 7% higher final accuracy. These differences in performance could tentatively be attributed to the high levels of education reported by participants in the current study: 92% had a university degree, which is in contrast to previous studies where the proportion was around 50% (e.g., Boissin et al., 2021, 2022; Franiatte et al., 2024).

A second thing to note is that all participants in the current study were adult volunteers, whereas many previous psychological training studies have primarily involved college students or paid online workers (e.g., Prolific or MTurk; see Barrett, 2020). Consequently, the more highly educated volunteers in our study might have shown higher levels of motivation and engagement during the experiment. At the same time, the fact that despite these variations in sample composition, we still observed similar overall training effects underscores the robustness of the training. Nevertheless, it may be worthwhile in future work to examine how individual differences could potentially account for variations in training accuracy. Against this backdrop, numerous studies pointed to the fact that the accuracy of both initial and final answers can be affected by individual differences in, among others, cognitive abilities (e.g., intelligence), thinking styles (e.g., the propensity for reflection), cultural backgrounds, age or education (e.g., Boissin et al., 2023b; Šrol & De Neys, 2021; Thompson & Johnson, 2014; Raoelison et al., 2021). It may be worthwhile in future work to examine whether and how these individual differences factors impact training efficiency.

In this study, we found that the debiasing training effect can be generalized to a language and cultural context different from the English-speaking one. It is worth noting that comparing studies conducted in different languages presents challenges, as there is no way to ensure complete similarity in stimuli and instructions between languages (Boroditsky, 2001). Note, however, that it was not our primary objective to contrast the precise extent of the training effect per se in this study. Instead, we mainly wanted to test whether the training can successfully debias people’s intuitive and deliberate reasoning in another language (i.e., whether there is a significant improvement to start with). Hence, empirically demonstrating that a debiasing training works, and impacts people’s intuitive reasoning is far from trivial in this respect.

However, it is also clear that the approach we introduced here can be further developed. Hence there are a number of limitations that one needs to take in mind. First, we only focused on (native) French speakers, who were tested with problem contents designed for a French-speaking test environment. Although this was an initial step towards opening up the training to more diversity, it is important to note that these participants are still considered a WEIRD population (i.e., Western Educated Industrialized Rich and Democratic societies, see Henrich et al., 2010). Ideally, future studies should also investigate the debiasing effect on other languages, cultural groups, or populations who live in conditions vastly different than the current group of French-speaking Europeans (e.g., Boissin et al., 2024; Trémolière et al., 2022).

Second, a critic might argue that the observed improvement in trained reasoners could be solely attributed to the general feedback on correct and incorrect responses, rather than the training per se. However, as Janssen et al. (2020) showed with the bat-and-ball task, providing minimal feedback to reasoners, on average, does not significantly impact accuracy. One explanation for this lies in the nature of errors in these reasoning tasks. Notably, prior studies indicated that biased reasoners often show minimal error sensitivity or bias detection from the onset (see De Neys, 2023, for a review). That is, even without feedback, people seem to implicitly detect that their answer is not fully warranted. This tentatively indicates that people are not biased because they do not realize that their response is incorrect but rather because they do not explicitly know how to arrive at the correct solution strategy, thereby arguing against a general feedback confound. As Janssen et al. (2020) put it, a more informative retrieval cue would be needed to arrive at the correct solution. Hence, if we want people to reason more accurately, it appears necessary to provide additional information about the correct solution strategy beyond simple feedback.

Third, one may also wonder whether our training results simply stem from a mere repetition effect. As we presented 48 problems in a row, it cannot be excluded that some reasoners benefited from spontaneous learning simply by repeatedly solving structurally similar problems. For instance, some reasoners in the control group improved “naturally” (from pre- to post-intervention, initial responses rose by 6.4 points and final responses rose by 1.4 points). However, this improvement is marginal and our key interest is the effect of the debiasing intervention on reasoning performance – which is much more pronounced in the training than in the control group. Especially, the Block x Group interaction effect is significant, indicating that there is an effect of the training but not for the control group. If the repeated exposure had led to a strong spontaneous learning effect, we should have observed a non-significant Block x Group interaction. Additionally, note that previous studies such as Raoelison and De Neys (2019) investigated how repeated exposure affects initial and final response accuracy (on the bat-and-ball task), and showed that even extensive repeated exposure has a limited impact on reasoners’ performance. Taken together, this seems to argue against a strong confounding spontaneous learning effect in our data.

Fourth, considering items of each task as random variables in our statistical model suggests that the training effect could readily generalize to other classic reasoning tasks. Although mastering these elementary logical principles is essential for sound reasoning, it’s important to note that these lab-based tasks remain somewhat artificial (e.g., Janssen et al., 2021; Politzer et al., 2017; Prado et al., 2020). Arguably, as highlighted in the introduction, biased judgments can have detrimental impacts across various domains of everyday life. Therefore, it will remain important to test the transition from laboratory conditions to more ecological settings. One could think here, for example, of more applied context such as classroom settings (e.g., Brault Foisy et al., 2015), medical diagnosis (e.g., Topol, 2024) or gender discrimination in recruitment decisions (e.g., Isaac et al., 2009).

Fifth, note also that additional methodologies such as mouse tracking can be considered to better understand the dynamics of reasoning and decision-making (e.g., Freeman & Ambady, 2010; Spivey et al., 2005). In particular, hand movements provide a constant flow of data that can reveal ongoing dynamics of processing with fine-grained temporal sensitivity (Freeman et al., 2011). It could help to further pinpoint the time course of fast ‘intuitive’ responses (Travers et al., 2016), and provide deeper insights into the cognitive processes underlying training effects.

Finally, one may wonder whether the training effect is sustainable over time. Here, for mere practical reasons (we recruited volunteers and tested them without assigning an identifier), we did not investigate the robustness of the training effect. However, previous debiasing studies (e.g., Boissin et al., 2021, 2022) conducted a retest two months after the initial training session. Results indicated that the training effect persisted – at least after two months – with accuracies after two months still being higher than before the initial training. Additionally, in a recent study (Franiatte et al., 2024), we found that the training effect could be boosted – and even made more robust over time – when participants take the training twice within a single week. Obviously, one could try to boost the training efficacy further with more immediate and/or frequent re-training. The optimal schedule remains to be explored here.

To conclude, in the present work, we replicated previous debiasing findings with a French-adapted training. This study suggests that simple interventions can be employed to boost sound reasoning – as early as the intuitive stage – in different parts of the world and confirms the suitability of the French versions for future research.

## Data Accessibility Statement

Raw data, analysis scripts, and preregistration for this study can be downloaded from our OSF page (https://osf.io/hk8rv/).

## Additional File

The additional file for this article can be found as follows:

10.5334/pb.1260.s1 Supplementary Material. Supplementary figures and tables cited in the main text are available here.

## Footnotes

After verification, one participant took the experiment in Québec, Canada. Since this individual identified as a native French speaker and their performance aligned with the overall trend, we decided not to exclude them from the analyses.
