# Supplementary material for: Adieu Bias: Debiasing Intuitions Among French Speakers
Source: Psychol Belg. 2024 Apr 18;64(1):42–57. doi: 10.5334/pb.1260 (PMC11025568; doi:10.5334/pb.1260)
Supplement: Supplementary Material. — Supplementary figures and tables cited in the main text are available here. [file pb-64-1-1260-s1.pdf]

Supplementary Material

A. Material: French items used in the study

BR = Base-Rate neglect, CF = Conjunction Fallacy, and BB = Bat-and-Ball tasks.

	Task	Conflict version	No-conflict version
1	BR	Cette étude concerne des boxeuses et des caissières de supermarché.<br>La personne 'W' est musclée.<br>Il y a 5 boxeuses et 995 caissières de supermarché.<br>Est-ce que la personne 'W' a plus de chance d'être :<br>• Une boxeuse<br>• Une caissière de supermarché	Cette étude concerne des boxeuses et des caissières de supermarché.<br>La personne 'W' est musclée.<br>Il y a 995 boxeuses et 5 caissières de supermarché.<br>Est-ce que la personne 'W' a plus de chance d'être :<br>• Une boxeuse<br>• Une caissière de supermarché
2	BR	Cette étude concerne des architectes et des chauffeurs de bus.<br>La personne 'C' est créative.<br>Il y a 6 architectes et 994 chauffeurs de bus.<br>Est-ce que la personne 'C' a plus de chance d'être :<br>• Un architecte<br>• Un chauffeur de bus	Cette étude concerne des architectes et des chauffeurs de bus.<br>La personne 'C' est créative.<br>Il y a 994 architectes et 6 chauffeurs de bus.<br>Est-ce que la personne 'C' a plus de chance d'être :<br>• Un architecte<br>• Un chauffeur de bus
3	BR	Cette étude concerne des écrivains et des ouvriers de chantier.<br>La personne 'F' est robuste.<br>Il y a 996 écrivains et 4 ouvriers de chantier.<br>Est-ce que la personne 'F' a plus de chance d'être :<br>• Un écrivain<br>• Un ouvrier de chantier	Cette étude concerne des écrivains et des ouvriers de chantier.<br>La personne 'F' est robuste.<br>Il y a 4 écrivains et 996 ouvriers de chantier.<br>Est-ce que la personne 'F' a plus de chance d'être :<br>• Un écrivain<br>• Un ouvrier de chantier
4	BR	Cette étude concerne des directeurs administratifs et des humoristes.<br>La personne 'K' est drôle.<br>Il y a 997 directeurs administratifs et 3 humoristes.<br>Est-ce que la personne 'K' a plus de chance d'être :<br>• Un directeur administratif<br>• Un humoriste	Cette étude concerne des directeurs administratifs et des humoristes.<br>La personne 'K' est drôle.<br>Il y a 3 directeurs administratifs et 997 humoristes.<br>Est-ce que la personne 'K' a plus de chance d'être :<br>• Un directeur administratif<br>• Un humoriste
5	BR	Cette étude concerne des hôtesses de l'air et des gardiens de prison.<br>La personne 'M' est charmante.<br>Il y a 3 hôtesses de l'air et 997 gardiens de prison. Est-ce que la personne 'M' a plus de chance d'être :<br>• Une hôtesse de l'air<br>• Un gardien de prison	Cette étude concerne des hôtesses de l'air et des gardiens de prison.<br>La personne 'M' est charmante.<br>Il y a 997 hôtesses de l'air et 3 gardiens de prison.<br>Est-ce que la personne 'M' a plus de chance d'être :<br>• Une hôtesse de l'air<br>• Un gardien de prison
6	BR	Cette étude concerne des pompiers et des riches héritiers.<br>La personne 'L' est courageuse.<br>Il y a 4 pompiers et 996 riches héritiers.<br>Est-ce que la personne 'L' a plus de chance d'être :<br>• Un pompier<br>• Un riche héritier	Cette étude concerne des pompiers et des riches héritiers.<br>La personne 'L' est courageuse.<br>Il y a 996 pompiers et 4 riches héritiers.<br>Est-ce que la personne 'L' a plus de chance d'être :<br>• Un pompier<br>• Un riche héritier
7	BR	Cette étude concerne des éboueurs et des hommes d'affaires.<br>La personne 'D' est ambitieuse.<br>Il y a 994 éboueurs et 6 hommes d'affaires.<br>Est-ce que la personne 'D' a plus de chance d'être :	Cette étude concerne des éboueurs et des hommes d'affaires.<br>La personne 'D' est ambitieuse.<br>Il y a 6 éboueurs et 994 hommes d'affaires.<br>Est-ce que la personne 'D' a plus de chance d'être :

		• Un éboueur<br>• Un homme d'affaire	• Un éboueur<br>• Un homme d'affaire
8	BR	Cette étude concerne des jardiniers et des PDG.<br>La personne 'S' est autoritaire.<br>Il y a 995 jardiniers et 5 PDG.<br>Est-ce que la personne 'S' a plus de chance d'être :<br>• Un jardinier<br>• Un PDG	Cette étude concerne des jardiniers et des PDG.<br>La personne 'S' est autoritaire.<br>Il y a 5 jardiniers et 995 PDG.<br>Est-ce que la personne 'S' a plus de chance d'être :<br>• Un jardinier<br>• Un PDG
9	BR	Cette étude concerne des agents immobiliers et des chômeurs.<br>La personne 'X' est illettrée.<br>Il y a 994 agents immobiliers et 6 chômeurs.<br>Est-ce que la personne 'X' a plus de chance d'être :<br>• Un agent immobilier<br>• Un chômeur	Cette étude concerne des agents immobiliers et des chômeurs.<br>La personne 'X' est illettrée.<br>Il y a 6 agents immobiliers et 994 chômeurs.<br>Est-ce que la personne 'X' a plus de chance d'être :<br>• Un agent immobilier<br>• Un chômeur
10	BR	Cette étude concerne des chirurgiennes et des adolescentes.<br>La personne 'V' est immature.<br>Il y a 995 chirurgiennes et 5 adolescentes.<br>Est-ce que la personne 'V' a plus de chance d'être :<br>• Une chirurgienne<br>• Une adolescente	Cette étude concerne des chirurgiennes et des adolescentes.<br>La personne 'V' est immature.<br>Il y a 5 chirurgiennes et 995 adolescentes.<br>Est-ce que la personne 'V' a plus de chance d'être :<br>• Une chirurgienne<br>• Une adolescente
11	BR	Cette étude concerne des dentistes et des profs de sport.<br>La personne 'J' est méticuleuse.<br>Il y a 3 dentistes et 997 profs de sport.<br>Est-ce que la personne 'J' a plus de chance d'être :<br>• Un dentiste<br>• Un prof de sport	Cette étude concerne des dentistes et des profs de sport.<br>La personne 'J' est méticuleuse.<br>Il y a 997 dentistes et 3 profs de sport.<br>Est-ce que la personne 'J' a plus de chance d'être :<br>• Un dentiste<br>• Un prof de sport
12	BR	Cette étude concerne des bibliothécaires et des DJ. La personne 'R' est calme.<br>Il y a 5 bibliothécaires et 995 DJ.<br>Est-ce que la personne 'R' a plus de chance d'être :<br>• Un bibliothécaire<br>• Un DJ	Cette étude concerne des bibliothécaires et des DJ.<br>La personne 'R' est calme.<br>Il y a 995 bibliothécaires et 5 DJ.<br>Est-ce que la personne 'R' a plus de chance d'être :<br>• Un bibliothécaire<br>Un DJ
13	BR	Cette étude concerne des nourrices et des femmes d'affaires.<br>La personne 'H' est attentionnée.<br>Il y a 4 nourrices et 996 femmes d'affaires.<br>Est-ce que la personne 'H' a plus de chance d'être :<br>• Une nourrice<br>Une femme d'affaire	Cette étude concerne des nourrices et des femmes d'affaires.<br>La personne 'H' est attentionnée.<br>Il y a 996 nourrices et 4 femmes d'affaires.<br>Est-ce que la personne 'H' a plus de chance d'être :<br>• Une nourrice<br>• Une femme d'affaire
14	BR	Cette étude concerne des scientifiques et des vendeuses par téléphone.<br>La personne 'E' est rigoureuse.<br>Il y a 6 scientifiques et 994 vendeuses par téléphone.<br>Est-ce que la personne 'E' a plus de chance d'être :<br>• Un scientifique<br>• Une vendeuse de téléphone	Cette étude concerne des scientifiques et des vendeuses par téléphone.<br>La personne 'E' est rigoureuse.<br>Il y a 994 scientifiques et 6 vendeuses par téléphone.<br>Est-ce que la personne 'E' a plus de chance d'être :<br>• Un scientifique<br>• Une vendeuse de téléphone
15	BR	Cette étude concerne des juges et des secrétaires. La personne 'T' est à l'écoute.<br>Il y a 996 juges et 4 secrétaires.<br>Est-ce que la personne 'T' a plus de chance d'être :<br>• Un juge<br>• Une secrétaire	Cette étude concerne des juges et des secrétaires. La personne 'T' est à l'écoute.<br>Il y a 4 juges et 996 secrétaires.<br>Est-ce que la personne 'T' a plus de chance d'être :<br>• Un juge<br>• Une secrétaire
16	BR	Cette étude concerne des procureures et des	Cette étude concerne des procureures et des

		infirmières.<br>La personne 'U' est rassurante.<br>Il y a 997 procureures et 3 infirmières.<br>Est-ce que la personne 'U' a plus de chance d'être :<br>• Une procureure<br>• Une infirmière	infirmières.<br>La personne 'U' est rassurante.<br>Il y a 3 procureures et 997 infirmières.<br>Est-ce que la personne 'U' a plus de chance d'être :<br>• Une procureure<br>• Une infirmière
17	CF	Serge, 25 ans, a étudié l'aérodynamique et aime les sports extrêmes.<br>Est-il plus probable que la personne décrite soit :<br>• Professeur d'histoire<br>• Croque-mort<br>• Professeur d'histoire et joueur de scrabble<br>• Professeur d'histoire et pilote de moto	Clara, 45 ans, a étudié l'aérodynamique et aime les sports extrêmes.<br>Est-il plus probable que la personne décrite soit :<br>• Pilote de moto<br>• Croque-mort<br>• Professeur d'histoire et joueur de scrabble<br>• Professeur d'histoire et pilote de moto
18	CF	Clara, 26 ans, a étudié le marketing web et aime les réseaux sociaux.<br>Est-il plus probable que la personne décrite soit :<br>• Gendarme<br>• Avaleur d'épée<br>• Gendarme et fan de puzzles<br>• Gendarme et youtubeur	Serge, 44 ans, a étudié le marketing web et aime les réseaux sociaux.<br>Est-il plus probable que la personne décrite soit :<br>• Youtubeur<br>• Avaleur d'épée<br>• Gendarme et fan de puzzles<br>• Gendarme et youtubeur
19	CF	Camille, 27 ans, a étudié la robotique et aime les Intelligences Artificielles.<br>Est-il plus probable que la personne décrite soit :<br>• Caissier<br>• Chanteur de pop international<br>• Caissier et cheerleader<br>• Caissier et hackeur	Lucas, 43 ans, a étudié la robotique et aime les Intelligences Artificielles.<br>Est-il plus probable que la personne décrite soit :<br>• Hackeur<br>• Chanteur de pop international<br>• Caissier et cheerleader<br>• Caissier et hackeur
20	CF	Lucas, 29 ans, a étudié la psychologie et aime les œuvres caritatives.<br>Est-il plus probable que la personne décrite soit :<br>• Huissier<br>• Charmeur de serpent<br>• Huissier et parieur sportif<br>• Huissier et bénévole	Camille, 41 ans, a étudié la psychologie et aime les œuvres caritatives.<br>Est-il plus probable que la personne décrite soit :<br>• Bénévole<br>• Charmeur de serpent<br>• Huissier et parieur sportif<br>• Huissier et bénévole
21	CF	Charles, 35 ans, a étudié la philosophie et aime la Grèce antique.<br>Est-il plus probable que la personne décrite soit :<br>• Coach sportif<br>• Eleveur d'otarie<br>• Coach sportif et fan de télé-réalité<br>• Coach sportif et collectionneur d'art	Chloé, 36 ans, a étudié la philosophie et aime la Grèce antique.<br>Est-il plus probable que la personne décrite soit :<br>• Collectionneur d'art<br>• Eleveur d'otarie<br>• Coach sportif et fan de télé-réalité<br>• Coach sportif et collectionneur d'art
22	CF	Mathieu, 39 ans, a étudié la comédie et aime rire.<br>Est-il plus probable que la personne décrite soit :<br>• Archiviste<br>• Directeur de banque<br>• Archiviste et karatéka<br>• Archiviste et clown	Manon, 33 ans, a étudié la comédie et aime rire.<br>Est-il plus probable que la personne décrite soit :<br>• Clown<br>• Directeur de banque<br>• Archiviste et karatéka<br>• Archiviste et clown
23	CF	Manon, 40 ans, a étudié l'immobilier et aime les objets de luxe.<br>Est-il plus probable que la personne décrite soit :<br>• Livreur de pizza<br>• Capitaine de sous-marin<br>• Livreur de pizza et maquilleur<br>• Livreur de pizza et collectionneur de montres	Mathieu, 32ans, a étudié l'immobilier et aime les objets de luxe.<br>Est-il plus probable que la personne décrite soit :<br>• Un collectionneur de montres<br>• Capitaine de sous-marin<br>• Livreur de pizza et maquilleur<br>• Livreur de pizza et collectionneur de montres

24	CF	David, 41 ans, a étudié l'art du cirque et aime la gymnastique.<br>Est-il plus probable que la personne décrite soit :<br>• Ramasseur de fruits<br>• Chef d'état<br>• Ramasseur de fruits et joueur de jeux vidéo<br>• Ramasseur de fruits et acrobate	Lucie, 31 ans, a étudié l'art du cirque et aime la gymnastique.<br>Est-il plus probable que la personne décrite soit :<br>• Acrobate<br>• Chef d'état<br>• Ramasseur de fruits et joueur de jeux vidéo<br>• Ramasseur de fruits et acrobate
25	CF	Steeve, 33 ans, a étudié la biologie et aime les balades en forêt.<br>Est-il plus probable que la personne décrite soit :<br>• Masseur<br>• Pilote de chasse<br>• Masseur et lutteur<br>• Masseur et ramasseur de champignon	Amélie, 37 ans, a étudié la biologie et aime les balades en forêt.<br>Est-il plus probable que la personne décrite soit :<br>• Ramasseur de champignon<br>• Pilote de chasse<br>• Masseur et lutteur<br>• Masseur et ramasseur de champignon
26	CF	Amélie, 32 ans, a étudié la théologie et aime les chants de chorale.<br>Est-il plus probable que la personne décrite soit :<br>• Travailleur dans un entrepôt<br>• Conducteur de Formule 1<br>• Travailleur dans un entrepôt et joueur de paintball<br>• Travailleur dans un entrepôt et chrétien	Steeve, 38 ans, a étudié la théologie et aime les chants de chorale.<br>Est-il plus probable que la personne décrite soit :<br>• Chrétien<br>• Conducteur de Formule 1<br>• Travailleur dans un entrepôt et joueur de paintball<br>• Travailleur dans un entrepôt et chrétien
27	CF	Antoine, 31 ans, a étudié l'informatique et aime les mangas.<br>Est-il plus probable que la personne décrite soit :<br>• Barman<br>• Diplomate<br>• Barman et fumeur de pipe<br>• Barman et joueur en ligne	Sophia, 39 ans, a étudié l'informatique et aime les mangas.<br>Est-il plus probable que la personne décrite soit :<br>• Joueur en ligne<br>• Diplomate<br>• Barman et fumeur de pipe<br>• Barman et joueur en ligne
28	CF	Sophia, 30 ans, a étudié l'économie et aime le bon tabac.<br>Est-il plus probable que la personne décrite soit :<br>• Assistant de vente<br>• Snowboardeur professionnel<br>• Assistant de vente et danseur de ballet<br>• Assistant de vente et fumeur de cigare	Antoine, 40 ans, a étudié l'économie et aime le bon tabac.<br>Est-il plus probable que la personne décrite soit :<br>• Fumeur de cigare<br>• Snowboardeur professionnel<br>• Assistant de vente et danseur de ballet<br>• Assistant de vente et fumeur de cigare
29	CF	Didier, 29 ans, a étudié l'ingénierie du son et aime les chaines hifis.<br>Est-il plus probable que la personne décrite soit :<br>• Boulanger<br>• Comte<br>• Boulanger et pratiquant de sports extrêmes<br>• Boulanger et fan de musique	Adèle, 29 ans, a étudié l'ingénierie du son et aime les chaines hifis.<br>Est-il plus probable que la personne décrite soit :<br>• Fan de musique<br>• Comte<br>• Boulanger et pratiquant de sports extrêmes<br>• Boulanger et fan de musique
30	CF	Adèle, 27 ans, a étudié le stylisme et aime la couture.<br>Est-il plus probable que la personne décrite soit :<br>• Aide-soignant<br>• Astronaute<br>• Aide-soignant et généalogiste<br>• Aide-soignant et passionné de mode	Didier, 43 ans, a étudié le stylisme et aime la couture.<br>Est-il plus probable que la personne décrite soit :<br>• Passionné de mode<br>• Astronaute<br>• Aide-soignant et généalogiste<br>• Aide-soignant et passionné de mode
31	CF	Samuel, 26 ans, a étudié les sciences de l'éducation et aime les enfants.<br>Est-il plus probable que la personne décrite soit :<br>• Agent de bord	Nelson, 44 ans, a étudié les sciences de l'éducation et aime les enfants.<br>Est-il plus probable que la personne décrite soit :<br>• Père au foyer

		• Duc<br>• Agent de bord et fan de courses de rallye<br>• Agent de bord et père au foyer	• Duc<br>• Agent de bord et fan de courses de rallye<br>• Agent de bord et père au foyer
32	CF	Julie, 34 ans, a étudié le féminisme et aime la musique hardcore.<br>Est-il plus probable que la personne décrite soit :<br>• Cordonnier<br>• Archevêque<br>• Cordonnier et témoin de Jéhovah<br>• Cordonnier et féministe	Nicolas, 35 ans, a étudié le féminisme et aime la musique hardcore.<br>Est-il plus probable que la personne décrite soit :<br>• Féministe<br>• Archevêque<br>• Cordonnier et témoin de Jéhovah<br>• Cordonnier et féministe
33	BB	Dans un supermarché, on peut acheter 320 tomates et avocats.<br>Il y a 300 tomates de plus que d'avocats. Combien y a-t-il d'avocats ?	Dans un supermarché, on peut acheter 160 tomates et avocats.<br>Il y a 100 tomates.<br>Combien y a-t-il d'avocats dans ce supermarché ?
34	BB	Dans une cuisine, il y a 260 couteaux et cuillères au total.<br>Il y a 200 couteaux de plus que de cuillères. Combien y a-t-il de cuillères ?	Dans une cuisine, il y a 220 couteaux et cuillères au total.<br>Il y a 200 couteaux.<br>Combien y a-t-il de cuillères dans la cuisine ?
35	BB	Un magasin de musique a 210 saxophones et flûtes au total.<br>Il y a 200 saxophones de plus que de flûtes. Combien y a-t-il de flûtes ?	Un magasin de musique a 270 saxophones et flûtes au total.<br>Il y a 200 saxophones.<br>Combien y a-t-il de flûtes dans le magasin ?
36	BB	Dans une entreprise, il y a 150 hommes et femmes au total.<br>Il y a 100 hommes de plus que de femmes. Combien y a-t-il de femmes ?	Dans une entreprise, il y a 330 hommes et femmes au total<br>Il y a 300 hommes.<br>Combien y a-t-il de femmes dans l'entreprise ?
37	BB	Un parc national a 650 roses et orchidées au total.<br>Il y a 600 roses de plus que d'orchidées.<br>Combien y a-t-il d'orchidées ?	Un parc national a 380 roses et orchidées au total.<br>Il y a 300 roses.<br>Combien y a-t-il d'orchidées dans le parc ?
38	BB	Dans une piscine, il y a 540 nageurs et plongeurs au total.<br>Il y a 500 nageurs de plus que de plongeurs. Combien y a-t-il de plongeurs ?	Dans une piscine, il y a 490 nageurs et plongeurs au total.<br>Il y a 400 nageurs.<br>Combien y a-t-il de plongeurs dans cette piscine ?
39	BB	Dans un magasin, il y a 480 clous et marteaux au total.<br>Il y a 400 clous de plus que de marteaux.<br>Combien y a-t-il de marteaux dans le magasin ?	Dans un magasin, il y a 550 clous et marteaux au total.<br>Il y a 500 clous.<br>Combien y a-t-il de marteaux dans le magasin ?
40	BB	Une ville possède 430 bus et trains au total.<br>Il y a 400 bus de plus que de trains.<br>Combien y a-t-il de trains dans la ville ?	Une ville possède 610 bus et trains au total.<br>Il y a 600 bus.<br>Combien y a-t-il de trains dans la ville ?
41	BB	Dans une forêt, il y a 640 chênes et érables au total.<br>Il y a 600 chênes de plus que d'érables.<br>Combien y a-t-il d'érables ?	Dans une forêt, il y a 390 chênes et érables au total.<br>Il y a 300 chênes.<br>Combien y a-t-il d'érables dans cette forêt ?
42	BB	Une entreprise emploie 580 techniciens et ingénieurs au total.<br>Il y a 500 techniciens de plus que d'ingénieurs.<br>Combien y a-t-il d'ingénieurs ?	Une entreprise emploie 450 techniciens et ingénieurs au total.<br>Il y a 400 techniciens.<br>Combien y a-t-il d'ingénieurs dans cette entreprise ?
43	BB	Pour un tournoi sportif, on a invité 530 joueurs et entraîneurs. Il y a 500 joueurs de plus que d'entraîneurs. Combien y a-t-il d'entraîneurs ?	Pour un tournoi sportif, on a invité 510 joueurs et entraîneurs. Il y a 500 joueurs. Combien y a-t-il d'entraîneurs invités à ce tournoi ?
44	BB	Sur une étagère, il y a 560 vis et tournevis au total. Il y a 500 vis de plus que de tournevis.<br>Combien y a-t-il de tournevis sur l'étagère ?	Sur une étagère, il y a 560 vis et tournevis au total.<br>Il y a 500 vis.<br>Combien y a-t-il de tournevis sur l'étagère ?
45	BB	Un directeur de magasin a acheté 310 bananes et kiwis.<br>Il y a 300 bananes de plus que de kiwis.	Un directeur de magasin a acheté 170 bananes et kiwis.<br>Il y a 100 bananes.

		Combien y a-t-il de kiwis ?	Combien y a-t-il de kiwis dans ce magasin ?
46	BB	Dans un restaurant, il y a 250 verres et tasses au total.<br>Il y a 200 verres de plus que de tasses.<br>Combien y a-t-il de tasses ?	Dans un restaurant, il y a 230 verres et tasses au total.<br>Il y a 200 verres.<br>Combien y a-t-il de tasses dans ce restaurant ?
47	BB	Un magasin met en exposition 190 pianos et harpes.<br>Il y a 100 pianos de plus que de harpes.<br>Combien y a-t-il de harpes ?	Un magasin met en exposition 280 pianos et harpes.<br>Il y a 200 pianos.<br>Combien y a-t-il de harpes dans ce magasin ?
48	BB	Dans un parc, il y a 140 adultes et enfants au total. Il y a 100 adultes de plus que d'enfants.<br>Combien y a-t-il d'enfants ?	Dans un parc, il y a 340 adultes et enfants au total.<br>Il y a 300 adultes.<br>Combien y a-t-il d'enfants ?

Note: For the base-rate task, we labelled the response that is in line with the base rates as the correct response. Critics of the base rate task (e.g., Barbey & Sloman, 2007; Gigerenzer et al., 1988) have long pointed out that if reasoners adopt a Bayesian approach and combine the base rate probabilities with the stereotypical description, this can lead to interpretative complications when the description is extremely diagnostic. For example, imagine that we have an item with males and females as the two groups and give the description that Person ‘A’ ‘is ‘pregnant’. Now, in this case, one would always need to conclude that Person ‘A’ is a woman, regardless of the base rates. The more moderate descriptions (such as ‘kind’ or ‘creative’) help to avoid this potential problem. In addition, the extreme base rates (i.e., 997/3, 996/4, 995/5) that were used in the current study further help to guarantee that even a very approximate Bayesian reasoner would need to pick the response cued by the base rates (see De Neys, 2014).

B. Conjunction fallacy problems: Frequency of each individual response option on conflict items

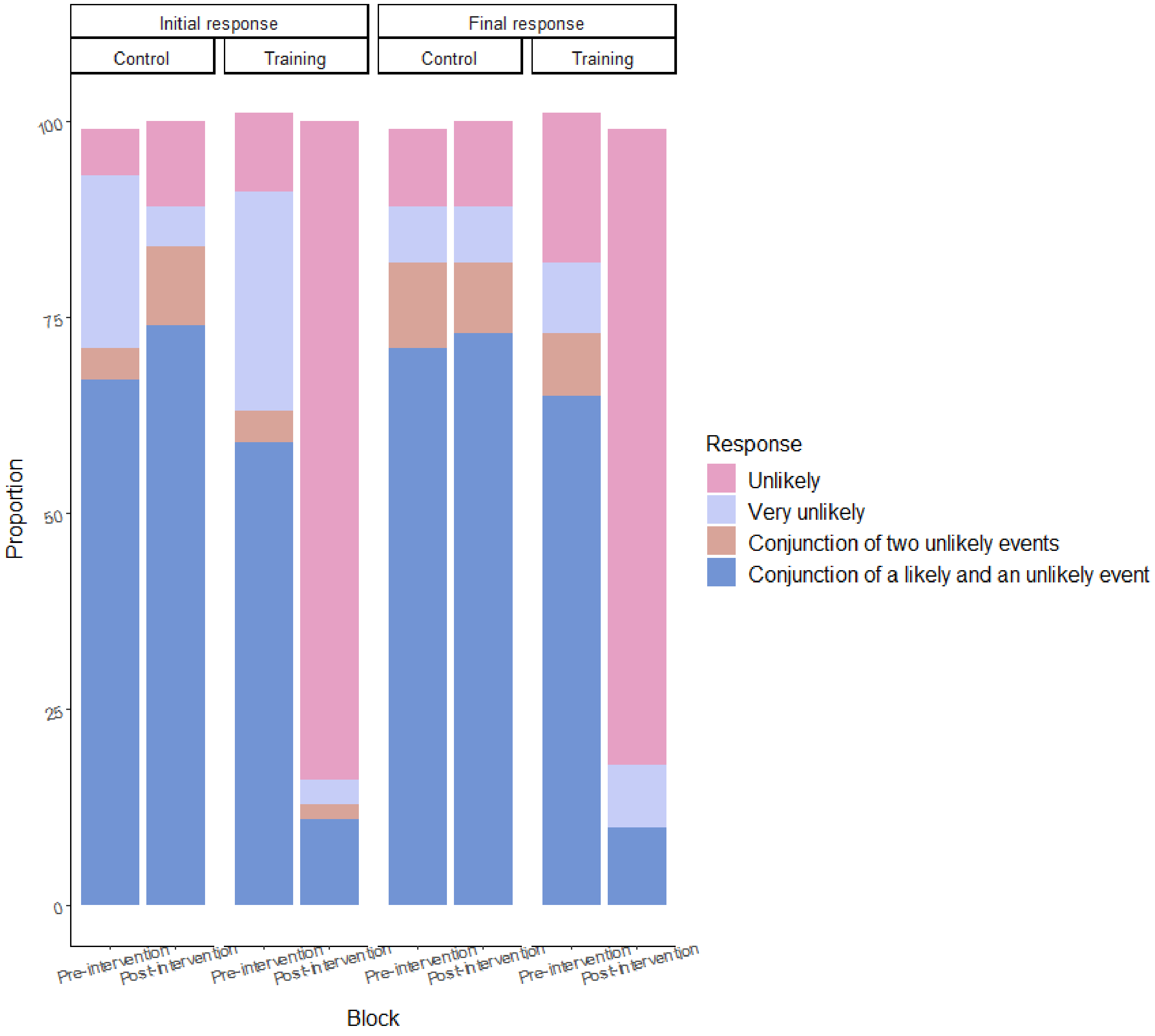

Figure S1. Frequency of each individual response option (conjunction fallacy conflict items) for the initial and the final responses, before and after the intervention in the control and training group.

C. Justification data

Table S1.

Frequency of different types of justifications for the final bat-and-ball (BB), base-rate (BR), conjunction fallacy (CF) conflict problems and all tasks combined (All) during the post-intervention of the study.

Task	Justification types	Control group		Training group	
		Correct response (n = 96)	*Incorrect response (n = 129)*	*Correct response (n = 170)*	*Incorrect response (n = 31)*
All	Math - Correct	64	3	105	2
	Math – Incorrect/Unspecified	7	45	4	9
	Guess	1	6	1	2
	Intuitions	13	53	30	13
	Other	11	22	30	5
BB	Math - Correct	35	3	43	-
	Math – Incorrect/Unspecified	-	18	-	6
	Guess	1	1	-	-
	Intuitions	3	8	7	5
	Other	-	3	4	2
BR	Math - Correct	25	-	37	1
	Math – Incorrect/Unspecified	7	4	1	1
	Guess	-	3	-	1
	Intuitions	9	13	8	3
	Other	10	6	14	-
CF	Math - Correct	4	-	25	1
	Math – Incorrect/Unspecified	-	23	3	2
	Guess	-	2	1	1
	Intuitions	1	32	15	5
	Other	1	13	12	3

Note: The coding format and procedure were based on Bago and De Neys (2019) for bat-and-ball, Boissin et al. (2022) for base-rate, and Franiatte et al. (2024) for conjunction fallacy tasks. A justification was considered correct when it explicitly mentioned the correct calculation for the bat-and-ball (e.g., ‘140 in total - 100 adults = 40 children / 2, the response is 20’) or the use of the base-rate (e.g., ‘Greater number of writers to constructions workers. For every 1 construction worker there are 249 writers, so the odds are stacked against it being a writer’) or when it explicitly referred to the conjunction principle (e.g., ‘There are always more people who are simply longshoreman than longshoreman and stargazer’). Other justifications, whether they mentioned an incorrect calculation or unspecified statement (e.g., ‘I did it in my head’) were coded as incorrect.

D. Confidence data

Note that due to a coding error the confidence data was not systematically recorded. The non-missing data is included in our data file on OSF, and an exploratory analysis of the partial data can be found below. We explored whether the training intervention affected biased reasoners' ability to detect conflict (i.e., conflict detection).

Table S2.

Conflict detection results on non-missing data. Percentage of mean difference in confidence ratings (SD) between initial correct no-conflict and initial incorrect conflict problems on each reasoning task: Bat-and-ball (BB), base-rate neglect (BR) and conjunction fallacy (CF).

Task	Group	Initial response - Session 1	
		Pre-intervention	Post-intervention
BB	Control	10.4 (24.8)	4.2 (19.9)
	Training	5.0 (23.3)	17.4 (33.3)
BR	Control	4.6 (21.4)	7.4 (17.4)
	Training	15.6 (36.8)	22.8 (32.7)
CF	Control	8.8 (16.2)	1.7 (20.8)
	Training	3.8 (13.8)	20.0 (27.4)

E. Bat-and-ball problems: Accuracy with and without reasoners who already knew the original bat-and-ball problem

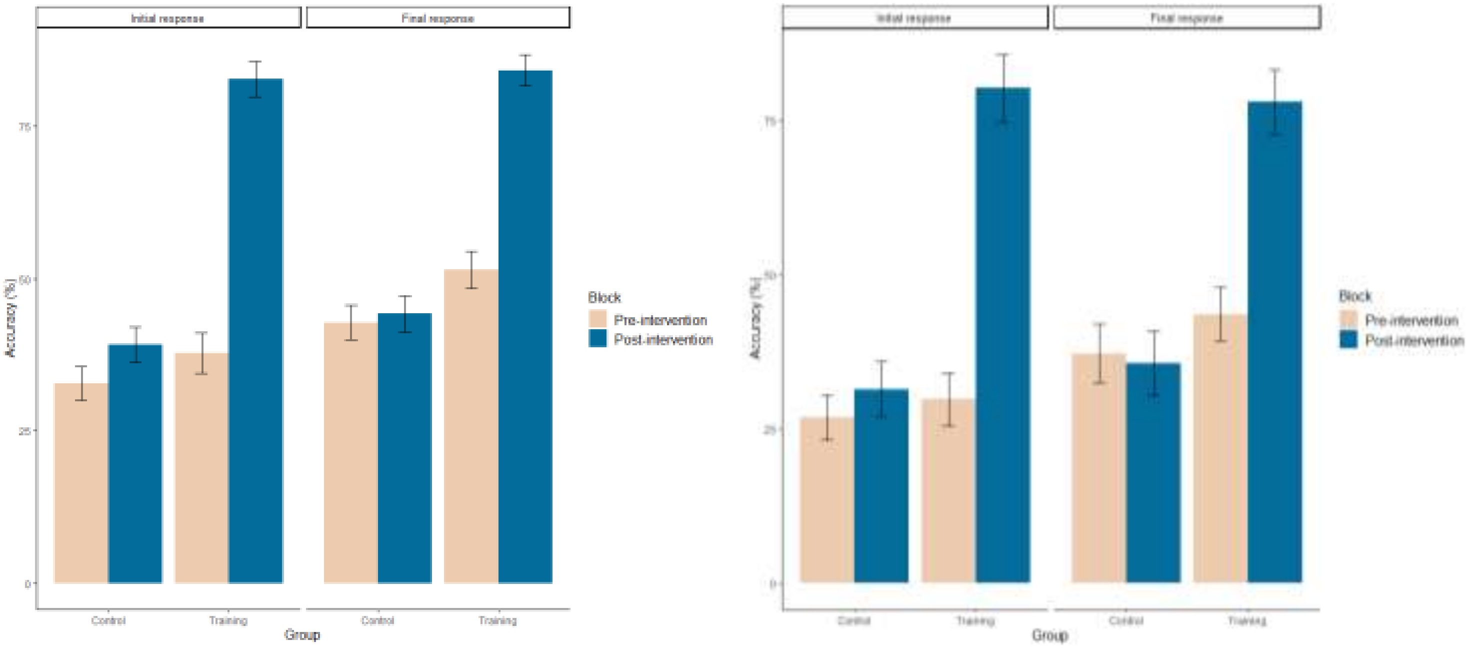

***Figure S2*.** Mean accuracy (%) of correct initial and final responses on conflict problems before and after the intervention, with (left panel) and without (right panel) reasoners who already knew the original bat-and-ball problem (Frederick, 2005). Error bars are standard errors. BB = bat-and-ball, BR = base-rate neglect, CF = conjunction fallacy tasks.

F. Accuracy for no-conflict problems

Table S3.

Average accuracy (%) for the no-conflict problems (SD) of bat-and-ball (BB), base-rate (BR) and conjunction fallacy (CF) tasks and combined (All task).

Task	Group	Initial response		Final response	
		Pre-intervention	Post-intervention	Pre-intervention	Post-intervention
BB	Control	78.8 (22.4)	96.1 (14.9)	82.6 (20.7)	97.4 (13.2)
	Training	79.5 (24.8)	91.0 (25.1)	84.6 (20.2)	93.5 (23.7)
BR	Control	95.7 (14.8)	94.0 (16.7)	98.3 (6.7)	95.4 (12.3)
	Training	87.9 (27.4)	95.3 (15.4)	93.6 (21.3)	96.6 (12.5)
CF	Control	83.8 (22.8)	73.4 (25.0)	85.6 (18.9)	73.1 (27.2)
	Training	86.7 (18.6)	94.0 (14.7)	86.0 (21.6)	93.2 (16.1)
All task	Control	85.9 (11.7)	87.7 (10.8)	88.3 (9.8)	88.5 (11.1)
	Training	84.3 (14.2)	93.5 (11.3)	87.7 (12.6)	94.4 (10.8)

G. Inferential statistics

Table S4.

Wald test results for the initial conflict responses, assessing the statistical significance of the fixed effect of the model with a 95% confidence interval. The significant p-values (*p* < .05) are marked with a *.

	Chi Square	Df	*p*
Group	1.43	1	.23
Block	4.18	1	.04*
Group:Block	103.16	1	<.001*

Table S5.

Wald test results for the final conflict responses, assessing the statistical significance of the fixed effect of the model with a 95% confidence interval. The significant p-values (*p* < .05) are marked with a *.

	Chi Square	Df	*p*
Group	3.59	1	.058
Block	0.19	1	.67
Group:Block	88.19	1	<.001*

H. Accuracy comparison in the current study (left panel) and in Franiatte et al.'s (2024) study (right panel)

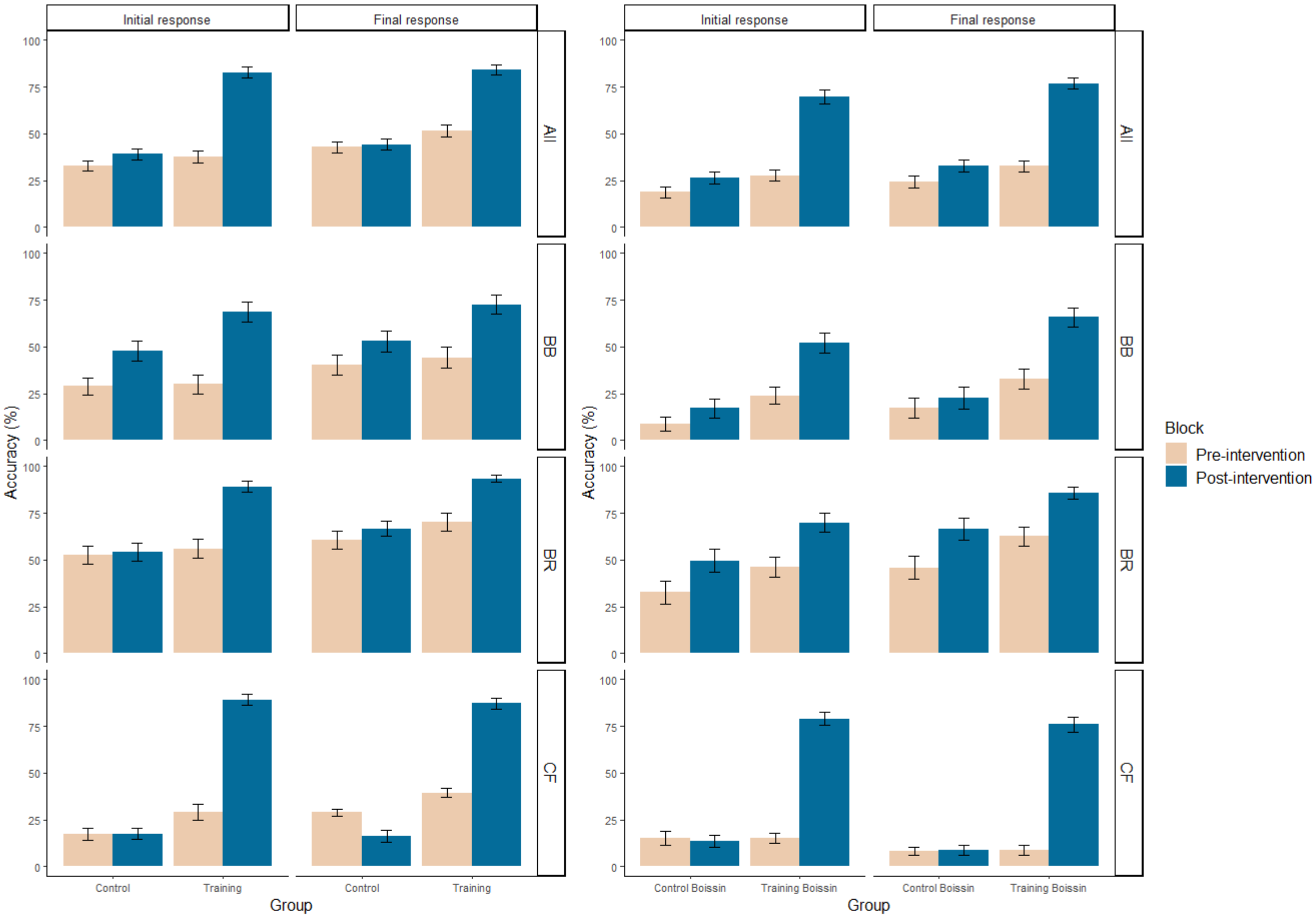

Figure S3. Comparison of mean accuracies (%) of correct initial and final responses on conflict problems for control and training groups, in the current study (left panel) and in Franiatte et al.'s (2024) study (right panel), for each task (BB, BR, CF), and combined (All). BB = bat-and-ball, BR = base-rate neglect, CF = conjunction fallacy, All = the composite mean across the three tasks.

Note. In Franiatte et al.'s study, the mean accuracies presented here corresponds to those of Session 1, Study 1.

I. Direction of change comparison with previous debiasing studies

***Table S6*.**

Comparison between proportions (%) of each direction of change (i.e., '00' trials, '01' trials, '10' trials and '11' trials) for the conflict problems (SD), in each block (pre- and post-intervention) and each group (control and training), in Franiatte et al.'s (2024) study and in the current study.

Direction	Group	Block	Direction of change in Franiatte et al. (2024)	Direction of change in the current study
00	Control	Pre-intervention	74.7 (28.3)	51.1 (26.1)
00	Control	Post-intervention	63.8 (23.3)	51.0 (27.1)
00	Training	Pre-intervention	64.7 (29.5)	43.0 (27.1)
00	Training	Post-intervention	19.0 (26.6)	11.5 (18.8)
01	Control	Pre-intervention	10.3 (16.5)	15.7 (12.1)
01	Control	Post-intervention	10.2 (16.2)	9.8 (11.9)
01	Training	Pre-intervention	9.1 (12.5)	19.2 (14.3)
01	Training	Post-intervention	11.6 (17.8)	5.7 (11.8)
11	Control	Pre-intervention	11.6 (20.4)	27.5 (24.1)
11	Control	Post-intervention	21.8 (21.3)	33.7 (25.1)
11	Training	Pre-intervention	22.6 (27.0)	32.2 (26.6)
11	Training	Post-intervention	64.2 (33.1)	79.1 (27.0)
10	Control	Pre-intervention	3.4 (8.4)	5.6 (8.5)
10	Control	Post-intervention	4.1 (7.3)	5.5 (7.2)
10	Training	Pre-intervention	3.6 (8.2)	5.6 (7.5)
10	Training	Post-intervention	5.2 (8.8)	3.6 (6.3)

Note. In Franiatte et al.'s study, the mean accuracies presented here corresponds to those of Session 1, Study 1.

J. Individual level direction of change

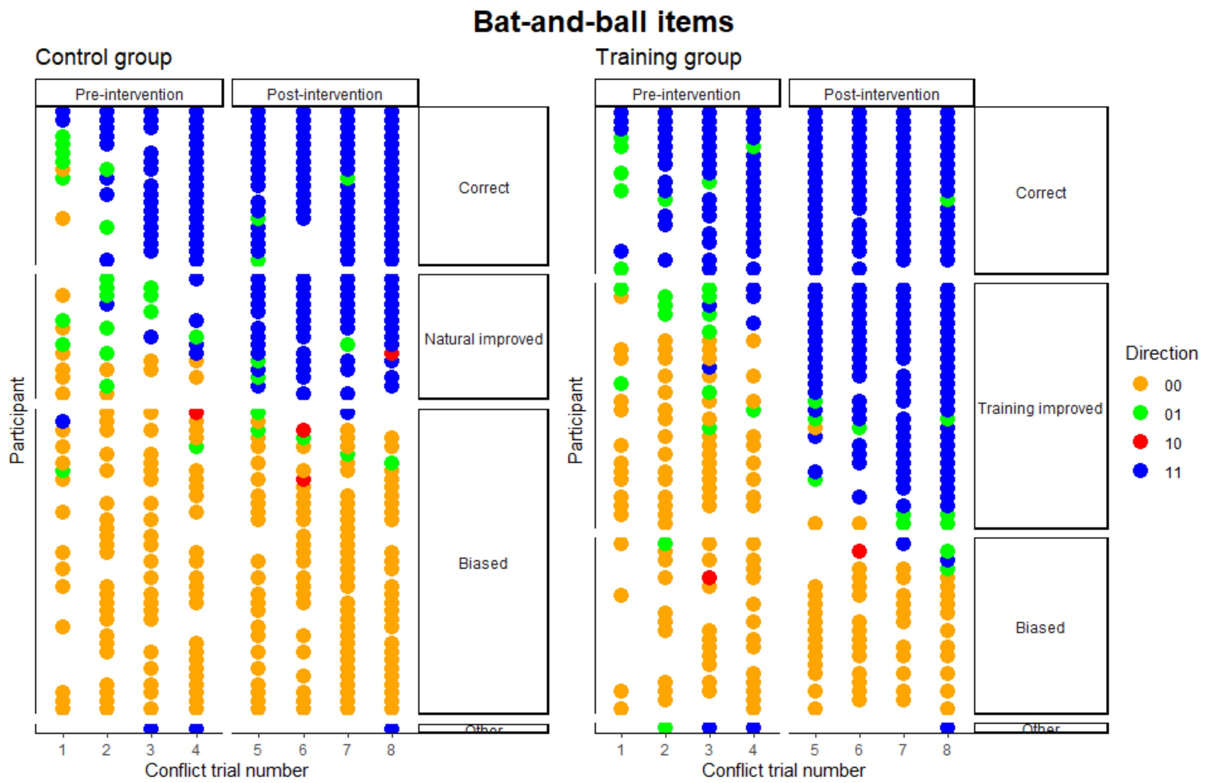

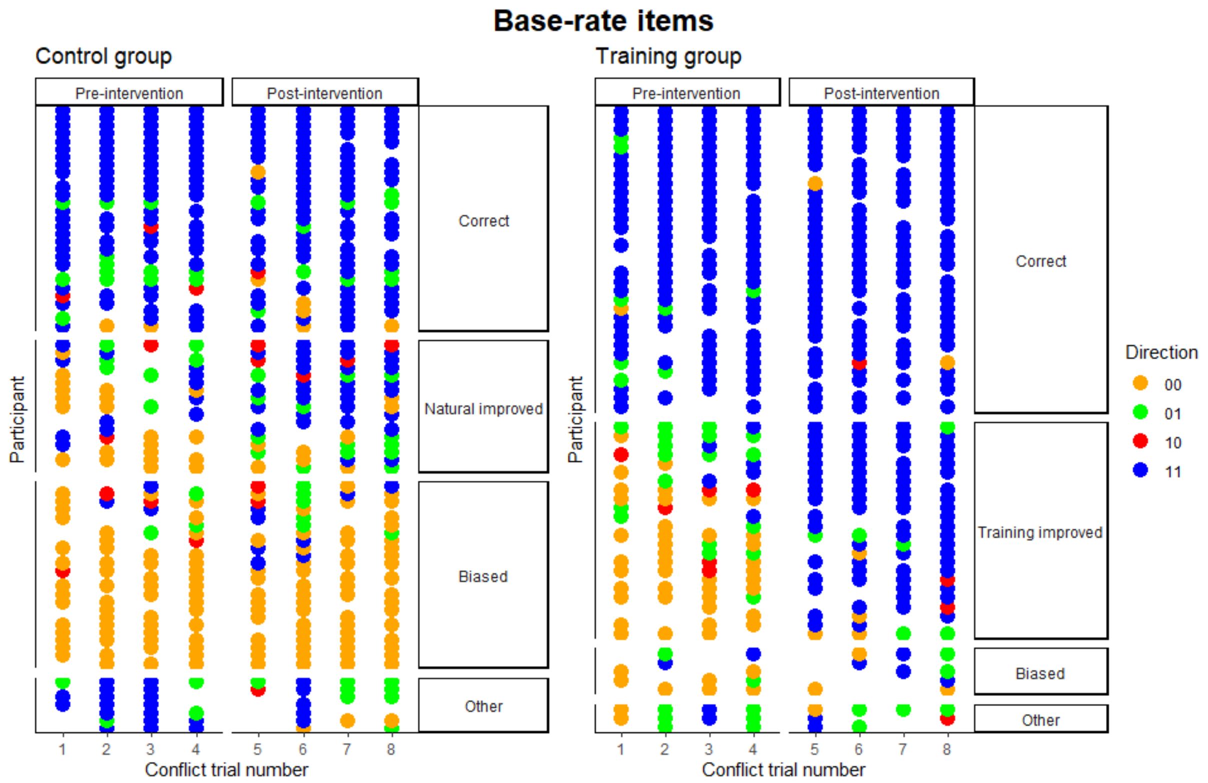

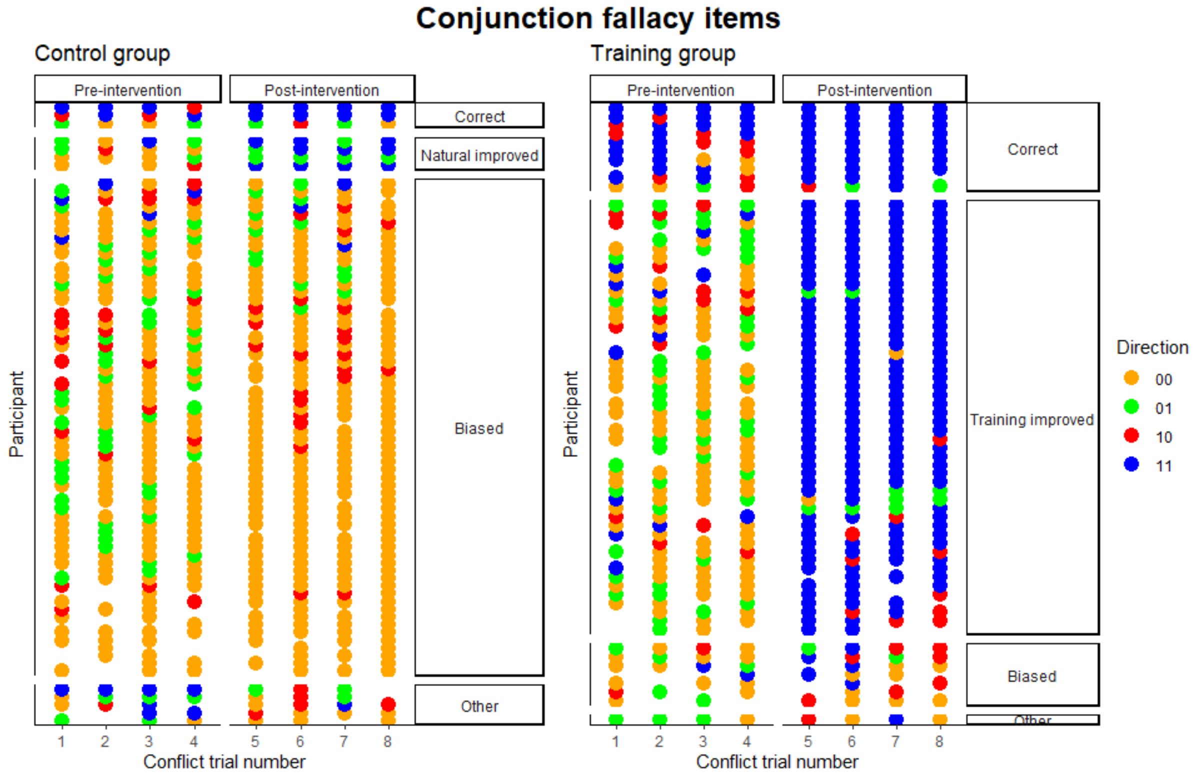

Figure S4. Individual level direction of change (each row represents one participant). Due to the exclusion of missed deadline and load trials (see Trial Exclusion), not all participants contributed 24 analysable trials.
